# Elucidation of the Mechanisms and Molecular Targets of *Sanhuang Xiexin* Decoction for Type 2 Diabetes Mellitus Based on Network Pharmacology

**DOI:** 10.1155/2020/5848497

**Published:** 2020-08-10

**Authors:** Manman Xu, Zhonghao Li, Lu Yang, Wujianwen Zhai, Nina Wei, Qiuyan Zhang, Bin Chao, Shijing Huang, Hanming Cui

**Affiliations:** ^1^Research and Development Center of Traditional Chinese Medicine, Guangan'men Hospital, China Academy of Chinese Medical Sciences, Beijing 100053, China; ^2^Department of Neurology, Dongfang Hosipital Beijing University of Chinese Medicine, Beijing 100078, China; ^3^Shaanxi University of Chinese Medicine, Department of Traditional Chinese Medicine, First Clinical Medical College, 712000 Shaanxi, China

## Abstract

*Sanhuang Xiexin* Decoction (SXD) is commonly used to treat type 2 diabetes mellitus (T2DM) in clinical practice of traditional Chinese medicine (TCM). In order to elucidate the specific analysis mechanisms of SXD for T2DM, the method of network pharmacology was applied to this article. First, the effective ingredients of SXD were obtained and their targets were identified based on the TCMSP database. The T2DM-related targets screened from the GEO database were also collected by comparing the differential expressed genes between T2DM patients and healthy individuals. Then, the common targets in SXD-treated T2DM were obtained by intersecting the putative targets of SXD and the differential expressed genes of T2DM. And the protein–protein interaction (PPI) network was established using the above common targets to screen key genes through protein interactions. Meanwhile, these common targets were used for GO and KEGG analyses to further elucidate how they exert antidiabetic effects. Finally, a gene pathway network was established to capture the core one in common targets enriched in the major pathways to further illustrate the role of specific genes. Based on the data obtained, a total of 67 active compounds and 906 targets of SXD were identified. Four thousand one hundred and seventy-six differentially expressed genes with a *P* value < 0.005 and ∣log2(fold change) | >0.5 were determined between T2DM patients and control groups. After further screening, thirty-seven common targets related to T2DM in SXD were finally identified. Through protein interactions, the top 5 genes (YWHAZ, HNRNPA1, HSPA8, HSP90AA1, and HSPA5) were identified. It was found that the functional annotations of target genes were associated with oxygen levels, protein kinase regulator, mitochondria, and so on. The top 20 pathways including the PI3K-Akt signaling pathway, cancers, HIF-1 signaling pathway, and JAK-STAT signaling pathway were significantly enriched. CDKN1A was shown to be the core gene in the gene-pathway network, and other several genes such as CCND1, ERBB2, RAF1, EGF, and VEGFA were the key genes for SXD against T2DM. Based on the network pharmacology approach, we identified key genes and pathways related to the prognosis and pathogenesis of T2DM and also provided a feasible method for further studying the chemical basis and pharmacology of SXD.

## 1. Introduction

Type 2 diabetes mellitus (T2DM) is currently one of the most important noncommunicable diseases that threaten human health in the current medical field due to its rapidly increasing morbidity and mortality. According to epidemiological surveys, the number of T2DM patients worldwide reached 415 million in 2015, of which 75% were in the developing countries. It is estimated that by 2040, the world will have nearly 642 million people with diabetes [1].With the growing research level of the pathological mechanisms of T2DM, plenty of pharmacological interventions targeting its mechanism of action have been widely used in the clinic to delay its progression [2]. The clinical characteristics of T2DM are mainly chronic glucose metabolism disorders. Its chronic hyperglycemia can cause long-term damage to various organs, which seriously affects the quality of life. However, most drugs for treating T2DM have certain adverse reactions, such as lactic acidosis, hypoglycemia, liver damage, and allergies, [3] which tend to be more evident with the time of treatment. In addition, the medical costs of T2DM and its complications also place a heavy financial burden on individuals, families, and governments. Thus, it has been the global research focus to search for more effective and safe drugs for the treatment of T2DM.

In recent years, with the continuous deepening of understanding and research on T2DM, traditional Chinese medicines (TCMs) have played a unique advantage in treating T2DM and its chronic complications, such as *Guizhi Shaoyao Zhimu* decoction [4], *Liuwei Dihuang* Pill [5], and *Shenqi Dihuang* decoction [6]. *Sanhuang Xiexin* decoction (SXD) is a classic prescription of three traditional herbal extracts including *Rheum palmatum* L. (*Dahuang*), *Scutellaria baicalensis* Georgi (*Huangqin*), and *Coptis chinensis* Franch. (*Huanglian*) at a ratio of 2 : 1 : 1, which has been used to cure diabetes since the Tang Dynasty (sixth century C.E.) [7]. In some clinical trials, SXD can not only significantly reduce fasting blood glucose and basal insulin dose [8] but also reduce weight and improve T2DM symptoms [9]. In addition, another clinical research conducted by Zhu et al. [10] found that the effective rates of improving insulin peripheral resistance and reducing blood sugar in the SXD group were 79.2% and 80.1%, mainly by means of reducing hyperinsulinemia and improving insulin sensitivity. Many research have reported that the major constituents of SXD includes berberine, palmatine, baicalin, baicalein, and coptisine, which can effectively exert bioactivities including anti-inflammation [11], gastric protection [12], antioxidation [13], antiatherosclerosis [14], and serum cholesterol reduction and benefits to early-stage diabetic nephropathy [15]. However, the possible molecular mechanism of the effective active ingredients in SXD against T2DM has been rarely studied, and the specific targets of exerting pharmacological effects are unknown, which becomes the main restriction on its wider application.

The TCM formula is usually composed of multiple drugs, so they have the characteristics of multicomponent, multipathway, and multitarget synergy [16]. However, it also brings the disadvantages of the specific single herb with unclear efficacy and unclear mechanisms of action due to this complexity, making it difficult for us to conduct a comprehensive and systematic study from the whole to the level of tissues, organs, cells, and molecules. With the rapid development of computational science, genomics, proteomics, and metabolomics, the concept of network pharmacology was presented to the public as a new interdisciplinary science, [17] which can reveal the effect mechanism of multimolecular drugs synergistically acting on the human body. In the present study, the network pharmacology approaches were used to elucidate the mechanisms and molecular targets of SXD for T2DM based on some data resources and tools.

## 2. Materials and Methods

### 2.1. Active Ingredient Screening

First, we used the Traditional Chinese Medicine Systems Pharmacology Database and Analysis Platform (TCMSP: https://sm.nwsuaf.edu.cn/lsp/tcmsp.php.) to screen effective compounds in SXD. At the same time, we use absorption, distribution, metabolism, and excretion (ADME) to select active ingredients with therapeutic effects, so we set the indicators as oral bioavailability (OB) ≥ 30% [18] and drug-likeness (DL) ≥0.18 [19]. TCMSP is currently one of the largest Chinese medicine databases, which consists of all the 499 Chinese herbs registered in the Chinese Pharmacopoeia with 29384 ingredients, 3311 targets, and 837 associated diseases [20]. And it is also a unique Chinese herbal medicine system pharmacology database which can acquire the herbs, chemicals, targets, and drug-target networks. Eventually, sixty- seven eligible compounds were obtained, 16 in Dahuang, 14 in Huanglian, and 37 in Huangqin.

### 2.2. Identify the Targets of the Active Ingredients

The DrugBank database [21] (https://www.drugbank.ca/) was used to identify the targets of the active ingredients in SXD. Two thousand four hundred and fifty-three targets were obtained from the database, 654 in *Dahuang*, 596 in *Huanglian*, and 1203 in *Huangqin*. Then, we continued to collect distinct targets related to the active ingredients in SXD after discarding duplicate data. Eventually, a total of 906 targets were collected after intersecting with the above 67 active ingredients, including 110 in *Dahuang*, 287 in *Huanglian*, and 509 in *Huangqin*.

### 2.3. Identify Targets for T2DM

Known differential expressed targets related to T2DM were acquired from the GEO database (https://www.ncbi.nlm.nih.gov/geo/, Series: GSE29221, Platform: GPL6947, 24 Samples), using “type 2 diabetes mellitus” as the keyword. Then, the genes with a *P* value < 0.05 and ∣log 2(fold change) | >0.5 were considered as target genes with significantly differential expression in T2DM patients.

### 2.4. Compound-Target Network Was Established

The common targets in SXD-treated T2DM were obtained by intersecting the putative targets of active compounds in SXD and the differential expressed genes of T2DM.To visualize the relationship between active compounds in SXD and the common targets, a compound-target network was built to reflect the graphical interactions using Cytoscape software [22] (version 3.7.1, Boston, MA, USA). The compound-target network consists of nodes and edges, in which nodes represent molecules that mainly refer to the compounds in SXD and the common targets, and edges indicate intermolecular interactions that mainly refer to the connections between compounds and targets.

### 2.5. PPI Network Was Established

The protein–protein interaction (PPI) network was constructed with the above common targets using the plugin Bisogenet [23] of Cytoscape 3.5.1 software. And the PPI data in the network came from the following databases: Biological General Repository for Interaction Datasets (GRID), Biological General Repository for Interaction Datasets (BioGRID), Human Protein Reference Database (HPRD), Biomolecular Interaction Network Database (BIND), Molecular Interaction Database (MINT), and Database of Interacting Proteins (DIP). To further screen out key targets that play an important role in the pathogenesis of T2DM, the nodes with topological importance in the PPI network were filtered through a series of parameters using Cytoscape plugin CytoNCA, such as degree centrality (DC), betweenness centrality (BC), closeness centrality (CC), eigenvector centrality (EC), local average connectivity-based method (LAC), and network centrality (NC). After a series of screening, we finally got a PPI network containing core genes, and on the other hand, it also showed the interaction between the important proteins.

### 2.6. GO and KEGG Analyses

Annotation, Visualization and Integrated Discovery database (DAVID, https://david.ncifcrf.gov, v6.8) [24] was used for GO analysis with biological processes, cellular components, and molecular functions. Among them, the functional categories with enriched genes (FDR < 0.05) were screened and then we select the top 20 GO functional categories for analysis. Similarly, the pathways with enriched genes (FDR < 0.05) were confirmed for further analysis using DAVID which assigned the Kyoto Encyclopedia of Genes and Genomes (KEGG) database.

### 2.7. Gene-Pathway Network Was Established

Based on the results of KEGG analysis, the enriched genes in the top 20 pathways were further selected for gene-pathway network analysis using Cytoscape software. This network can intuitively show the relationship between the genes and pathways, and screen for key genes that play an important role in the process of T2DM. Finally, the gene-pathway network was constructed, in which the size of nodes represents the topological importance of genes and pathways.

## 3. Results

### 3.1. Putative Targets of SXD and T2DM Analysis

**Figure 1:**
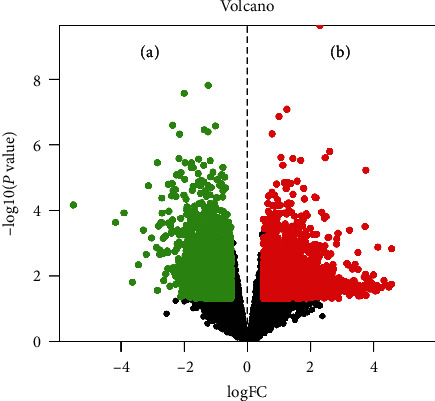
Volcano map of the differential genes. The abscissa represents logFC, which is the fold change in the gene expression. The ordinate represents -log10(*P* value), which is the statistical significance of the change in gene expression. The green dots on (a) represent downregulated genes, and the red dots on (b) represent upregulated genes.

Through the screening of the TCMSP database, 67 SXD compounds which meet the requirements were finally selected as the candidate compounds (Table 1). From the GEO database, 25136 T2DM-related targets were acquired, which proved to be involved in the pathological process of T2DM. According to the obtained differential expression genes, a volcano map was drawn to better show the distribution of genes, in which there were red upregulated and green downregulated dots. As shown in Figure 1, we obtained a total of 4176 differential expressed genes, including 2271 downregulated genes and 1905 upregulated genes.

### 3.2. Compound-Target Network Analysis

**Figure 2:**
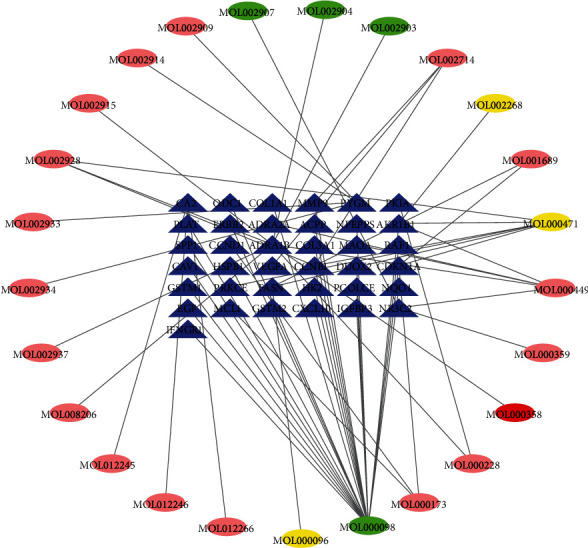
Compound-target network: the blue triangles represent targets related to T2DM in SXD; the yellow, green, and pink ovals represent the compounds from *Dahuang*, *Huanglian*, and *Huangqin*, respectively. The red oval represents a common component from *Dahuang* and *Huangqin*.

Through the intersection between the putative targets of active compounds in SXD and the differential expressed genes of T2DM, 37 common targets were identified. Through screening and removal of duplicates, there are 25 compounds related to T2DM in SXD, of which there are 3 compounds in *Dahuang*, 4 in *Huanglian*, 17 in *Huangqin*, and a common compound from *Dahuang* and *Huangqin*. As shown in Figure 2, the compound-target network was established with the 37 common targets using Cytoscape software. The network contained 62 nodes (25 compounds in SXD and 37 common targets) and 66 edges which indicated the compound-target interactions. According to the degree, the compounds with the largest three ranking values in SXD were MOL000098 quercetin in *Huanglian*, MOL000449 aloe-emodin in *Dahuang*, and MOL000471 stigmasterol in *Huangqin*, respectively. In addition, the OB of quercetin, aloe-emodin, and stigmasterol is 46.43, 83.38, and 43.83%, respectively. Therefore, the three compounds may be key active ingredients of SXD due to their important position in the compound-target network.

### 3.3. PPI Network Analysis

**Figure 3:**
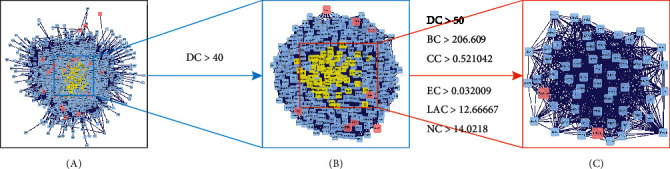
PPI network. (a) The combined PPI network of SXD-related targets and T2DM-related targets. (b) PPI network with important targets extracted from (a). (c) PPI network with core targets extracted from (b).

The PPI network is a relational network of biomolecules, and it plays an important role in the biological processes. Therefore, if the functional modules in the PPI network were identified, it could help understand the mechanism of biological activity and the pathogenesis of diseases [25]. Therefore, a PPI network of the common targets was constructed using PPI data. As shown in Figure 3(a), the PPI network consisted of 1859 nodes and 33869 edges, which represented 1859 interacting protein and 33869 interactions. According to the previous research, when the degree of all nodes was 40, which was twice as large as the median degree, targets with significant functions can be screened [26]. Then, as shown in Figure 3(b), the further filtered PPI network consisting of 521 nodes and 15710 edges was constructed. In this network, the median parameters of DC, BC, CC, EC, LAC, and NC were 50, 206.609, 0.521042, 0.032009, 12.66667, and 14.0218, respectively. The above data can be used as new reference standards for further screening, so the targets were determined with DC > 50, BC > 206.609, CC > 0.521042, EC > 0.032009, LAC > 12.66667, and NC > 14.0218 (Figure 3(c)). After a series of topology analysis, a total of 65 core target genes were eventually confirmed for SXD against T2DM, which are likely to exert antidiabetic mechanisms through direct and indirect effects.

### 3.4. GO and KEGG Analyses

**Figure 4:**
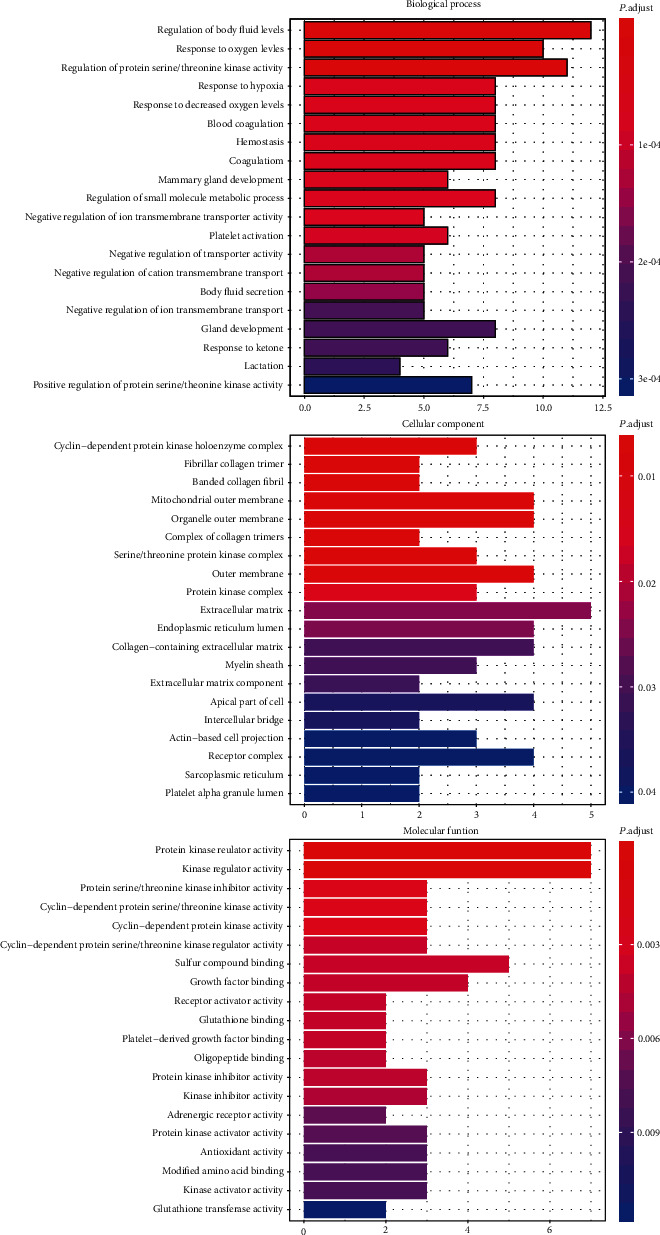
GO enrichment analysis. The top 20 GO enriched terms with FDR < 0.05 were screened. The abscissa represents the *P* adjust value of the enriched terms and the ordinate represents the name of the enriched terms.

A total of the 37 common targets in SXD-treated T2DM obtained from the compound-target network (Figure 2) were used to perform GO and KEGG pathway analyses. And the DAVID database was used during the data processing. Among them, the candidate targets for GO enrichment analysis were carried out in three different biological aspects, namely, biological process, cellular component, and molecular function. There were 172 enrichment GO terms with FDR < 0.05, including 387 in biological process, 23 in cellular component, and 40 in molecular function. The top 20 terms among the enriched entries were shown in Figure 4, of which there are 26 genes in biological process, 25 genes in cellular component, and 21 genes in molecular function. Through the analysis of enriched GO items, these genes distributed in biological processes, cellular components, and molecular functions were mainly manifested in the regulation of oxygen levels, regulation of serine/threonine kinase activity, mitochondria, platelet activation, and protein kinase regulator activity.

**Figure 5:**
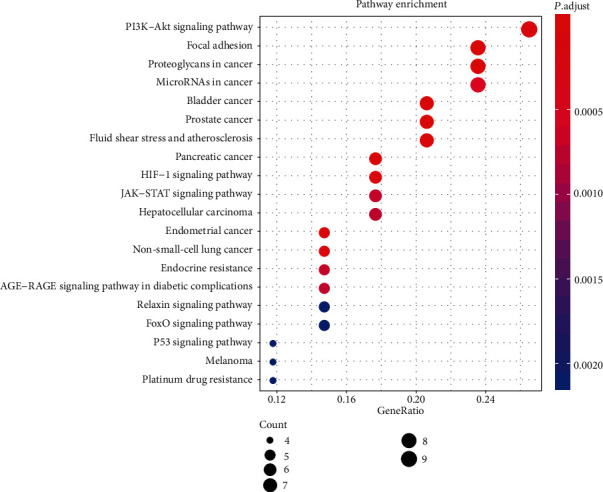
KEGG pathway enrichment analysis. The top 20 significant pathways with FDR < 0.05 were identified. The abscissa represents the GeneRatio of the enriched genes and the ordinate represents the name of the enriched pathways. The size of the dots represents the number of genes, and the color represents the significance of the FDR value.

Similarly, we also performed KEGG analysis to determine which pathways exert an enormous function on the pathological mechanism. As shown in Figure 5, the top 20 significantly enriched pathways (FDR < 0.05) were identified, in which there were PI3K-Akt signaling pathway, cancers, HIF-1 signaling pathway, JAK-STAT signaling pathway, endocrine resistance, and AGE-RAGE signaling pathway in diabetic complications.

### 3.5. Gene-Pathway Network Analysis

**Figure 6:**
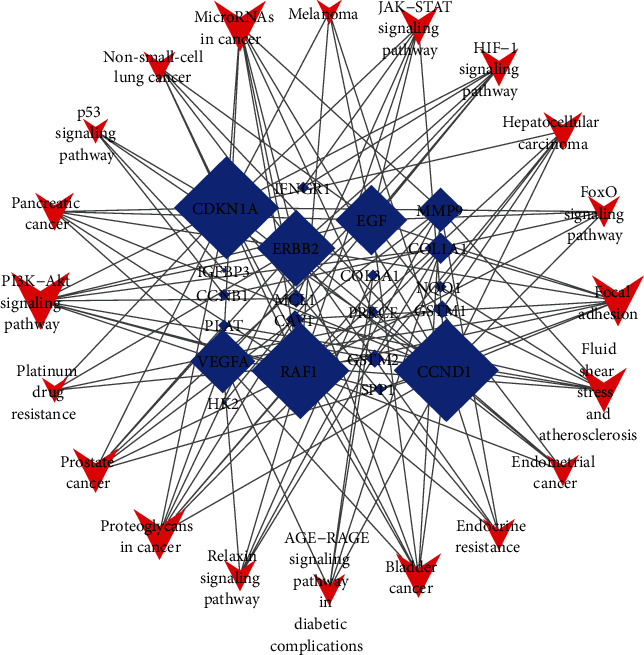
Gene-pathway network. Degree centrality (DC) was used to perform the topological analysis of the 20 pathways and 21 genes. The blue diamonds represent candidate genes and the red V-shapes represent pathways. The size of nodes represents the value of degree centrality.

In order to further screen the key genes enriched in the significant pathways, a gene-pathway network was established using Cytoscape software (Figure 6). Degree centrality (DC) was used to perform the topological analysis of the twenty pathways and twenty-one genes obtained in the KEGG analysis (Figure 5). In the gene-pathway network, the V shapes represent pathways and the diamond shapes represent the screened target genes. Through the visualized network, it can be intuitively seen that CDKN1A had the largest DC and was the core gene in the entire diagram. At the same time, several other genes, such as CCND1, RAF1, ERBB2, EGF, and VEGFA, also had larger DC. And all of them were the key target genes for SXD against T2DM.

## 4. Discussion

T2DM is an endocrine disease with clinical manifestations of metabolic disorders and chronic complications, and its occurrence is related to a variety of factors such as heredity, lifestyle, biochemistry, posture, and environment. The pathogenesis of T2DM mainly involves islet *β*-cell dysfunction, insulin resistance, inflammatory response, and oxidative stress response. SXD was an effective common prescription in the treatment of T2DM in TCM, which has demonstrated significant clinical effects in TCM clinical practice. According to previous studies, *Huanglian*, *Huangqin*, and *Dahuang* three components in SXD have antidiabetic effects. The active compounds in *Huanglian* can improve glucose metabolism, pancreatic beta cells, and insulin resistance and modulate the gut microbiota [27]. The active compounds in *Huangqin* can improve glucose metabolism and insulin sensitivity in peripheral tissue [28]. The active compounds in *Dahuang* can improve glucose metabolism and *β*-cell function, modulate the gut microbiota, and reduce oxidative stress and inflammation [29, 30]. However, it is not clear how SXD works in the antidiabetic pathological processes. Therefore, we use the method of network pharmacology to clarify the mechanism of active ingredients in SXD acting on T2DM.

In this paper, the compound-target network suggested that some compounds of SXD affected most targets. For instance, quercetin, aloe-emodin, and stigmasterol acted on 25, 6, and 5 targets, respectively. Consequently, they may probably be vital multifunctional active compounds of SXD against T2DM. It is well known that both quercetin [31–33] and aloe-emodin [34] exhibit a wide range of biological effects, including anti-inflammatory, antioxidant, anticancer, and especially the protective effect on T2DM. Youl et al. [35] reported that quercetin could significantly increase insulin secretion via ERK1/2 phosphorylation in INS-1 cells. And previous reports indicate that quercetin was able to promote wound healing of T2DM rats and improved glycemia by modulating inflammation and oxidative stress [36]. Besides, studies have shown that aloe-emodin can effectively treat *β*-cell failure in T2DM patients, because it can prevent the occurrence of pancreatic *β*-cell sugar glucotoxicity by regulating proinflammatory cytokines (IFN-*γ*, and IL1*β*) [37]. Similarly, stigmasterol is a member of the plant sterol family and has multiple pharmacological effects, such as antiosteoarthritis, anti-inflammatory, and antitumor properties. [38–41] One recent study showed that an extract from banana containing 21.91% stigmasterol exhibited a potential antidiabetic effect in alloxan-induced diabetic rats [42]. It can be seen that quercetin, aloe-emodin, and stigmasterol have significant antidiabetic effects in T2DM. And they all have high oral bioavailability and are considered to be representative compounds in SXD.

As shown in Figure 3, in order to get more accurate genes related to T2DM, a series of parameters were performed to screen for the key targets in the PPI network. Eventually, 65 significant targets were determined, which may play an important role in the pathophysiology of T2DM through direct or indirect effects. Among these genes, the top 5 genes were YWHAZ, HNRNPA1, HSPA8, HSP90AA1, and HSPA5, respectively. One research reported that YWHAZ mRNA and protein levels are significantly upregulated in the placental tissues of gestational diabetes mellitus (GDM) patients due to the downregulation of miR-214 [43]. The result indicates that elevated expression of YWHAZ protein played a regulatory role in the occurrence of GDM. HNRNPA1 maintains the stability of blood glucose in T2DM by regulating the sensitivity of insulin in muscle cells. A study by Zhao et al. [44]. showed that the metabolic capacity and insulin sensitivity of patients with T2DM are worsened by reduced expression of skeletal muscle HNRNPA1 because it inhibits glycogen synthesis in the body.

Heat-shock proteins (HSPs) can protect cells from oxidative stress, inflammation, and apoptosis because they can counteract the deleterious effects of hyperglycemia in the target organs of diabetes vascular complications [45]. Changes in the expression of related HSPs (HSPA8 [46], HSP90AA1 [47], and HSPA5 [48],) have been confirmed in complications of diabetes and are functionally related to hyperglycemia-induced cell damage. Therefore, the above five genes may have the potential to serve as valuable clinical biomarkers and provide new directions for the treatment of T2DM.

The common targets of SXD against T2DM were used to perform biological processes, cellular components, and molecular function analysis. In the biological processes, there are enriched terms of regulation of body fluid level, response to oxygen levels, regulation of protein serine/threonine kinase activity, hypoxia, and platelet activation (Figure 4). Different levels of body fluid level may play an important role in determining morphologic features that appear in diabetic macular edema (DME). Studies have pointed out that body fluid levels are related to the severity of diabetic retinopathy (DR) and that volume overload is an independent risk factor for it [49]. Patients with T2DM are easily prone to be in a state of hypoxia because of the increase in glycated hemoglobin levels which result in the weakened capacity of releasing oxygen into the plasma. And a study of Mason et al. found evidence that skeletal muscle oxygenation during exercise was more impaired in T2DM patients than in overweight and sedentary controls [50–53]. Protein kinases also exhibited an important role in the progression of T2DM. Some data suggest that STK11 can improve the lipotoxicity of islet cells and the glucose regulation of insulin secretion by activating the LKB1-AMPK pathway, regulating the secretion of insulin-like growth factor 1 (IGF-1) to improve IR in T2DM patients [54–56]. In addition, due to the unique inflammatory environment under diabetic condition, platelets are more prone to adhesion to endothelial cells and can be used as a sensitive biomarker for T2DM with vascular diseases [57]. Besides, these biological processes were also related to cellular components and molecular function, including collagen trimer, mitochondrial, endoplasmic reticulum, growth factor, and glutathione binding. As shown by some studies, ultrastructural abnormalities in cells of T2DM patients have also been found, such as cytoplasmic vacuolization, mitochondrial swelling, abnormal chromatin condensation, and endoplasmic reticulum expansion.

In the KEGG analysis, the top 20 KEGG pathways were significantly enriched including the PI3K-Akt signaling pathway, cancers, HIF-1 signaling pathway, JAK-STAT signaling pathway, endocrine resistance, and AGE-RAGE signaling pathway in diabetic complications. The PI3K-Akt signaling pathway was the most prominently enriched term. In T2DM, insulin is the major ligand that regulates metabolism in the PI3K/AKT pathway. Thus, the PI3K-Akt signaling pathway is activated by secreted insulin after eating, which increases glucose utilization and reduces gluconeogenesis in the liver and muscle [58]. Besides, the PI3K-Akt signaling can also exhibit its importance by mediating growth factor signals in glucose homeostasis, lipid metabolism, and protein synthesis [59]. Hypoxia is a pathological state in the cells of T2DM patients, and the HIF-1 signaling pathway plays a complex role in this process [60]. There is one clue that HIF1*α* activation can exacerbate the inflammation and fibrosis status of T2DM during hypoxia, thereby elevating obesity and insulin resistance in mice [61]. The JAK/STAT pathway is known to be involved in the inflammatory response and is one of the key ways of cytokine signal transduction. Inhibition of JAK phosphorylation and activation can prevent phosphorylation and activation of STATs, thereby alleviating insulin resistance because of reduced production of cytokines [62]. Studies have shown that the deficiency of STAT4 in high-fat diet-fed mice in T2DM mice was associated with reduced adipose tissue inflammation and decreased insulin resistance [63]. In this study, several pathways related to cancer were also significantly enriched (Figure 5). The association between T2DM and cancer may be explained in part by some shared risk factors such as aging, obesity, and diet [64]. In addition, the notion that glucose metabolism and cancer are associated can also be speculated in relation to the antidiabetes drug metformin, which has reported associations with reduced risk of cancer [65]. In addition, SXD may function by regulating other pathways, including endocrine resistance, AGE-RAGE signaling pathway, FoxO signaling pathway, and P53 signaling pathway. Among these, the FoxO pathway is one of those key factors in the transition from IR to the damage of islet *β*-cells [66].

Gene-pathway network was established to investigate core target genes for SXD against T2DM. The network showed that CDKN1A had the largest DC, which may be the core target gene. Other top five genes were selected as key target genes, namely, CCND1, RAF1, ERBB2, EGF, and VEGFA. CDKN1A is a substance induced by tumor suppressor P53, which is needed in G1-S blocking of DNA damaged cells. Its main role is to prevent apoptosis, maintain beta cell quality, and participate in DNA repair after DNA damage [67]. Studies of gene sequencing from human islets with and without T2DM have shown that both CDKN1A and CCND1 are highly expressed in diabetic patients [68].. RAF1 kinase specifically works to control cell proliferation to maintain the quantity of pancreatic beta cells [69]. In T2DM patients, lower RAF1 kinase levels were found to have a negative effect on the proliferation of pancreatic beta cell [70–72]. ERBB2 has shown close associations with molecules governing lipid metabolism [73] and impaired glucose metabolism in T2DM [74]. Additionally, a cross-sectional study by Memon et al. [75] showed a significant association between ERBB2 and hyperglycemia and insulin resistance. EGFR can be abnormally activated during diabetes or atherosclerosis [76] and the enhanced EGFR activity with downstream endoplasmic reticulum (ER) stress in cardiovascular system has been reported in T2DM [77, 78]. VEGFA is found to be most relevant to diabetic retinopathy (DR) in the VEGF family. Xu et al. [79] have found that upregulated VEGFA can promote the development of DR via the low expression of microRNA-15b in retinal capillary endothelial cells and pericytes of diabetic rats.

## 5. Conclusion

In this study, the network pharmacological approaches were used to elucidate the mechanisms and molecular targets of SXD on T2DM. Quercetin, aloe-emodin, and stigmasterol in SXD can regulate most targets related to T2DM. Through the PPI network screening, it was found that 5 key genes (YWHAZ, HNRNPA1, HSPA8, HSP90AA1, and HSPA5) obtained through protein interactions may provide new ideas for the treatment of diabetes. SXD may regulate an antidiabetic function through the specific biological processes including regulation of body fluid level, response to oxygen levels, regulation of protein serine/threonine kinase activity, hypoxia, and platelet activation. The above functions are mainly performed through the PI3K-AKT signaling pathway, HIF-1 signaling pathway, JAK-STAT signaling pathway, and some other cancer pathways. And CDKN1A, CCND1, RAF1, ERBB2, EGF, and VEGFA in common targets were the key targets of active components in SXD against T2DM. Although we have tried to study the role of SXD in the treatment of T2DM through network pharmacology as far as possible, further experiments in vivo and in vitro are still needed to validate these views.

## Figures and Tables

**Table 1:** Active compounds and ADME parameters of *Sanhuang Xiexin* decoction (SXD).

ID	Name	OB	DL	Source	ID	Name	OB	DL	Source
MOL002235	Eupation	50.8	0.41	*Dahuang*	MOL002908	5,8,2′-Trihydroxy-7-methoxyflavone	37.01	0.27	*Huangqin*
MOL002251	Mutatochrome	48.64	0.61	*Dahuang*	MOL002909	5,7,2,5-Tetrahydroxy-8,6-dimethoxyflavone	33.82	0.45	*Huangqin*
MOL002259	Physciondiglucoside	41.65	0.63	*Dahuang*	MOL002910	Carthamidin	41.15	0.24	*Huangqin*
MOL002260	Procyanidin B-5,3′-O-gallate	31.99	0.32	*Dahuang*	MOL002911	2,6,2′,4′-Tetrahydroxy-6′-methoxychaleone	69.04	0.22	*Huangqin*
MOL002268	Rhein	47.07	0.28	*Dahuang*	MOL002913	Dihydrobaicalin_qt	40.04	0.21	*Huangqin*
MOL002276	Sennoside E_qt	50.69	0.61	*Dahuang*	MOL002914	Eriodyctiol (flavanone)	41.35	0.24	*Huangqin*
MOL002280	Torachrysone-8-O-beta-D-(6′-oxayl)-glucoside	43.02	0.74	*Dahuang*	MOL002915	Salvigenin	49.07	0.33	*Huangqin*
MOL002281	Toralactone	46.46	0.24	*Dahuang*	MOL002917	5,2′,6′-Trihydroxy-7,8-dimethoxyflavone	45.05	0.33	*Huangqin*
MOL002288	Emodin-1-O-beta-D-glucopyranoside	44.81	0.8	*Dahuang*	MOL002925	5,7,2′,6′-Tetrahydroxyflavone	37.01	0.24	*Huangqin*
MOL002293	Sennoside D_qt	61.06	0.61	*Dahuang*	MOL002926	Dihydrooroxylin A	38.72	0.23	*Huangqin*
MOL002297	Daucosterol_qt	35.89	0.7	*Dahuang*	MOL002927	Skullcapflavone II	69.51	0.44	*Huangqin*
MOL002303	Palmidin A	32.45	0.65	*Dahuang*	MOL002928	Oroxylin a	41.37	0.23	*Huangqin*
MOL000358	Beta-sitosterol	36.91	0.75	*Dahuang*	MOL002932	Panicolin	76.26	0.29	*Huangqin*
MOL000471	Aloe-emodin	83.38	0.24	*Dahuang*	MOL002933	5,7,4′-Trihydroxy-8-methoxyflavone	36.56	0.27	*Huangqin*
MOL000554	Gallic acid-3-O-(6'-O-galloyl)-glucoside	30.25	0.67	*Dahuang*	MOL002934	Neobaicalein	104.34	0.44	*Huangqin*
MOL000096	(-)-Catechin	49.68	0.24	*Dahuang*	MOL002937	Dihydrooroxylin	66.06	0.23	*Huangqin*
MOL001454	Berberine	36.86	0.78	*Huanglian*	MOL000358	Beta-sitosterol	36.91	0.75	*Huangqin*
MOL013352	Obacunone	43.29	0.77	*Huanglian*	MOL000359	Sitosterol	36.91	0.75	*Huangqin*
MOL002894	Berberrubine	35.74	0.73	*Huanglian*	MOL000525	Norwogonin	39.4	0.21	*Huangqin*
MOL002897	Epiberberine	43.09	0.78	*Huanglian*	MOL000552	5,2′-Dihydroxy-6,7,8-trimethoxyflavone	31.71	0.35	*Huangqin*
MOL002903	(R)-canadine	55.37	0.77	*Huanglian*	MOL000073	Ent-epicatechin	48.96	0.24	*Huangqin*
MOL002904	Berlambine	36.68	0.82	*Huanglian*	MOL000449	Stigmasterol	43.83	0.76	*Huangqin*
MOL002907	Corchoroside A_qt	104.95	0.78	*Huanglian*	MOL001458	Coptisine	30.67	0.86	*Huangqin*
MOL000622	Magnograndiolide	63.71	0.19	*Huanglian*	MOL001490	bis[(2S)-2-Ethylhexyl] benzene-1,2-dicarboxylate	43.59	0.35	*Huangqin*
MOL000762	Palmidin A	35.36	0.65	*Huanglian*	MOL001506	Supraene	33.55	0.42	*Huangqin*
MOL000785	Palmatine	64.6	0.65	*Huanglian*	MOL002879	Diop	43.59	0.39	*Huangqin*
MOL000098	Quercetin	46.43	0.28	*Huanglian*	MOL002897	Epiberberine	43.09	0.78	*Huangqin*
MOL001458	Coptisine	30.67	0.86	*Huanglian*	MOL008206	Moslosooflavone	44.09	0.25	*Huangqin*
MOL002668	Worenine	45.83	0.87	*Huanglian*	MOL010415	11,13-Eicosadienoic acid, methyl ester	39.28	0.23	*Huangqin*
MOL008647	Moupinamide	86.71	0.26	*Huanglian*	MOL012245	5,7,4′-Trihydroxy-6-methoxyflavanone	36.63	0.27	*Huangqin*
MOL001689	Acacetin	34.97	0.24	*Huangqin*	MOL012246	5,7,4′-Trihydroxy-8-methoxyflavanone	74.24	0.26	*Huangqin*
MOL000173	Wogonin	30.68	0.23	*Huangqin*	MOL012266	Rivularin	37.94	0.37	*Huangqin*
MOL000228	(2R)-7-Hydroxy-5-methoxy-2-phenylchroman-4-one	55.23	0.2	*Huangqin*	MOL002935	Baicalin	29.53	0.77	*Huangqin*
MOL002714	Baicalein	33.52	0.21	*Huangqin*					

## Data Availability

My data is obtained through authoritative database and software analysis. I can guarantee its reliability.
